# 1470 nm Diode Laser Enucleation *vs* Plasmakinetic Resection of the Prostate for Benign Prostatic Hyperplasia: A Randomized Study

**DOI:** 10.1089/end.2018.0499

**Published:** 2019-03-12

**Authors:** Jun Zhang, Xilong Wang, Yanbin Zhang, Chaoliang Shi, Minqi Tu, Guowei Shi

**Affiliations:** Department of Urology, The Fifth People's Hospital of Shanghai, Fudan University, Shanghai, P.R. China.

**Keywords:** benign prostatic hyperplasia, 1470 nm diode laser, enucleation, TURP

## Abstract

***Objective:*** The purpose of the current work was to comparatively assess 1470 nm diode laser enucleation of the prostate (DiLEP) and plasmakinetic resection of the prostate (PKRP) for treating benign prostatic hyperplasia (BPH).

***Patients and Methods:*** From January 2016 to March 2017, 157 individuals with bladder outflow obstruction caused by BPH were randomized to DiLEP and PKRP groups, for prospective analysis. Of these, 152 cases were evaluated before operation and at 3, 6, and 12 months postsurgery. Patient baseline properties, presurgery data, and postsurgical outcomes were comparatively assessed, as well as complications.

***Results:*** There were no significant preoperative differences between surgical groups. DiLEP-treated cases showed remarkable reduced operative time, postsurgical bladder irrigation time, catheterization duration, and hospital stay compared with the PKRP group (*P* < 0.001). Hemoglobin amount decrease was markedly less pronounced after DiLEP (*P* = 0.004). However, no patients needed blood transfusion in either group. The decrease in sodium level showed no marked differences between the DiLEP and PKRP groups (*P* = 0.380). In addition, complications were comparable and no significant differences in both groups. At 3, 6, and 12 months, International Prostate Symptom Score (IPSS), quality of life (QoL), maximum flow rate (Qmax), and postvoid residual (PVR) were similar in both groups (*P* > 0.05).

***Conclusions:*** DiLEP and PKRP are similar in efficacy and safety for relieving obstruction and low urinary tract symptoms. Compared with PKRP, DiLEP has decreased risk of hemorrhage, operative time, bladder irrigation time, catheterization duration, and hospital stay. However, IPSS, QoL, Qmax, and PVR were similar for both procedures within 12 postoperative months.

## Introduction

Benign prostatic hyperplasia (BPH) constitutes the major etiology of lower urinary tract symptoms (LUTS) in male individuals aged >50 years. Transurethral resection of the prostate (TURP) represents the gold standard in the operative management of BPH, with demonstrated safety, efficacy, and durability.^1^ Although important technologic advances in the last few decades have decreased surgery-related undesirable events, complications, including bleeding (0.3%), capsular perforation (0.1%), transfusion (2%), and transurethral resection syndrome (TURS; 0.8%), remain a great concern.^2^ Therefore, a novel minimally invasive technology has been proposed, with laser surgery considered the new standard.^3^

The first laser enucleation of the prostate for BPH was described by Fraundorfer and Gilling in 1998.^4^ Then, a variety of laser types, for example, holmium, thulium, potassium-titanyl-phosphate (KTP), and diode lasers, have been used for treating BPH.^5–8^ The first diode laser had approval from the U.S. Food and Drug Administration (FDA) in 2007, with wide use thanks to remarkable tissue vaporization capacity and a great coagulation property.^9^ This laser operated at 1470 nm absorbed by both water and hemoglobin. In addition, the 1470 nm diode laser remarkably improves International Prostate Symptom Score (IPSS), quality of life (QoL), maximum flow rate (Qmax), and postvoid residual (PVR).^10^

To assess the 1470 nm diode laser enucleation of the prostate (DiLEP) for BPH, a prospective randomized clinical trial (RCT) with 12-month follow-up was carried out, with plasmakinetic bipolar resection of the prostate (PKRP) as a control procedure.

## Patients and Methods

### Patients

**FIG. 1.**
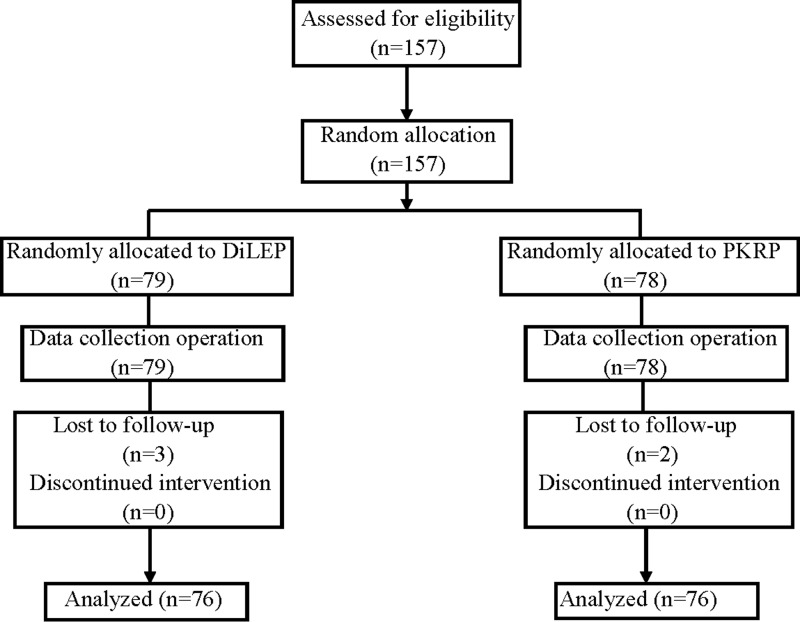
In all, 157 cases were randomized to the DiLEP (79) and PKRP (78) groups after ethics committee approval and written informed consent from patients. Finally, 152 patients (DiLEP 76 *vs* PKRP 76) with complete follow-up data were analyzed. DiLEP = diode laser enucleation of the prostate; PKRP = plasmakinetic resection of the prostate.

The current prospective, single-blinded RCT was conducted between January 2016 and March 2017 in our department; all cases with LUTS due to BPH with indication^11^ for endosurgical treatment were invited to participate in this clinical study. In all, 157 cases were randomized to the DiLEP (79) and PKRP (78) groups after ethics committee approval and written informed consent from patients. Grouping strategy was performed with sequential numbering and sealed envelopes. The patients were assigned envelopes by a computerized random number generator. Cases eligible for surgical treatment of BPH, with a prostate volume less than or equal to 80 mL, were included. Exclusion criteria were as follows: neurogenic bladder, urethral stricture, prostate carcinoma, and a history of urethral or prostate surgery. Finally, 152 patients (DiLEP 76 *vs* PKRP 76) with complete follow-up data were analyzed (Fig. 1).

### Assessment parameters

All patients underwent urine analysis, digital rectal examination, serum prostate-specific antigen (PSA) level evaluation, and urodynamic examination. The prostate was assessed for size by transrectal ultrasonography (TRUS), with the volume obtained as height × length × width × π/6. If prostate cancer was suspected, TRUS-guided biopsy was carried out for confirmation. Patient baseline properties were obtained, including age, prostate volume, PSA, Qmax, PVR, IPSS, and the QoL. Hemoglobin and serum sodium were measured before and 2 hours postsurgery. Perioperative indexes as well as peri- and postsurgical complications were recorded, for example, operative time, serum sodium and hemoglobin level changes, postsurgery irrigation time, catheterization time, hospitalization duration, blood transfusion requirement, TURS, capsular perforation, and urethral stricture. Follow-up was performed at 3, 6, and 12 postoperative months. Clinical outcomes, including IPSS, QoL, Qmax and PVR, were assessed at each follow-up.

### Instruments and surgical procedures

All operative procedures were carried out by one chief surgeon experienced in DiLEP and PKRP. The patients underwent detailed preoperative risk evaluation and were administered epidural or general anesthesia for surgical procedures in the lithotomy position. Major equipment for surgery included the following: Gyrus Plasmakinetic superpulse system generator (Gyrus), 120 W 1470 nm diode semiconductor laser generator (Miracle Laser, Wuhan, China), Olympus 12° resectoscope (Olympus), and Hawk 30° laser operator and morcellator system (Hawk, Hangzhou, China).

### Surgical technique

The generator settings for PKRP were 180 and 100 W for cutting and coagulation, respectively. Physiologic saline was used for irrigation. In the PKRP group, bipolar TURP was performed routinely using a 26F Olympus continuous irrigation resectoscope. Incision depth was close to the surgical capsule.

**FIG. 2.**
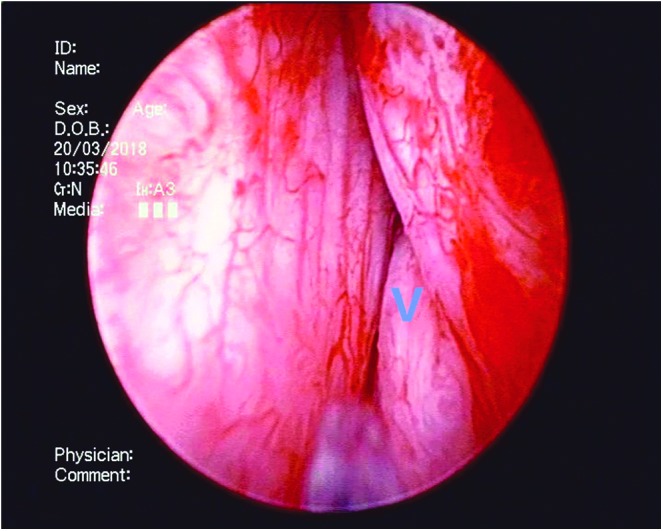
The resectoscope is placed into the bladder (B) to observe the urethra and estimate the range between bladder neck and verumontanum (V). The ureteral orifices and the shape of the prostate are also assessed.

**FIG. 3.**
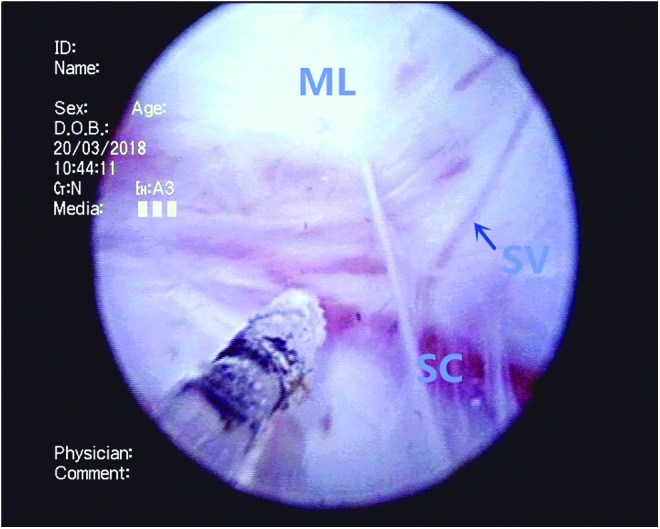
The middle lobe (ML) is enucleated from the surgical capsule (SC) by the diode laser. Denuded supply vessels (SV) on the capsule surface are identified. In case of bleeding, the laser beam is redirected to the specific area for hemostasis.

**FIG. 4.**
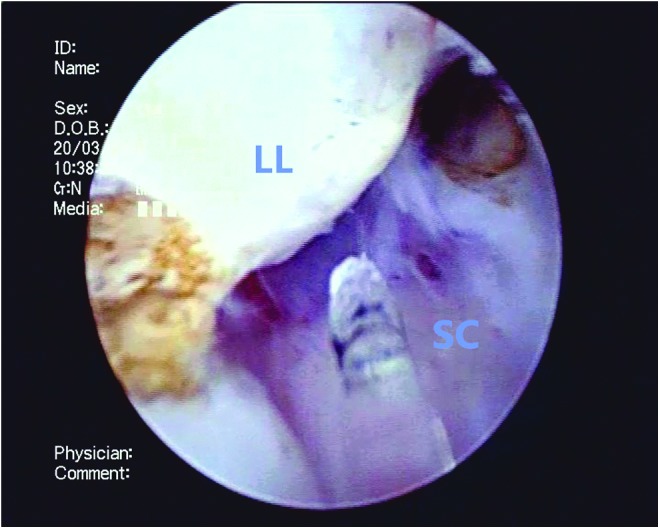
The left lateral lobe (LL) is similarly enucleated along the surgical capsule, moving in a counterclockwise position.

**FIG. 5.**
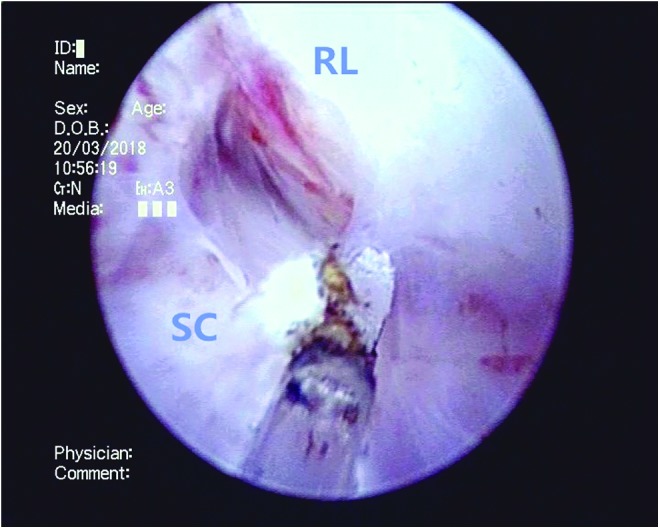
The right lateral lobe (RL) is also similarly enucleated along the surgical capsule, moving in a clockwise position.

**FIG. 6.**
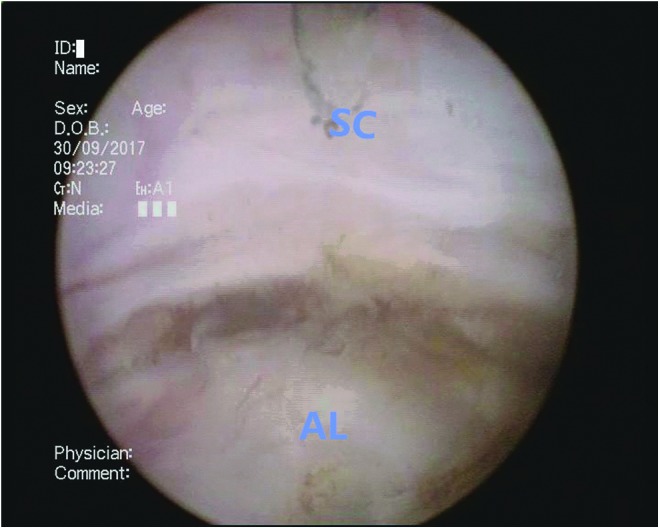
The anterior lobe (AL) is enucleated along the surgical capsule at the bladder neck.

**FIG. 7.**
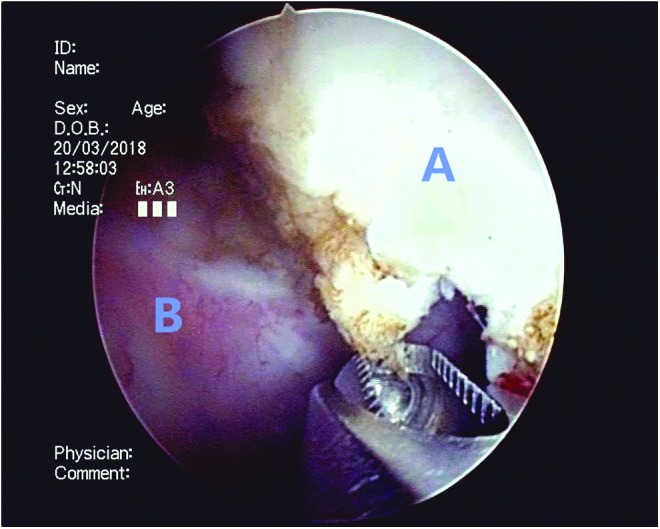
The adenomas (A) in the bladder were removed by the morcellator system.

The 1470 nm diode laser generator for DiLEP had settings of 120 and 30 W for vaporization and coagulation, respectively. A 26F resectoscope was inserted in the bladder under video assistance by an endosurgical device. The incision was started proximal to the verumontanum to the bladder neck at 5 o′ clock and 7 o′ clock positions, with the urethral mucosa incised to surgical capsule's level. The middle lobe was enucleated retrogradely off the bladder neck. The lateral lobes were similarly enucleated along the capsule, moving clockwise (right lateral lobe) or counterclockwise (left lateral lobe). In case of bleeding, the laser beam was redirected to the specific area for hemostasis with 30 W power. Finally, the adenomas in the bladder were removed by the morcellator system (Figs. 2–7). Complete enucleation should not be performed in all cases; patients with a small prostate gland were treated by vaporesection. Therefore, a combination of enucleation and vaporesection was performed. At the end of both procedures, a 22F three-way Foley catheter was inserted, and physiologic saline irrigation was continually administered.

### Statistical analysis

All measurement data are mean ± standard deviation, and were assessed by Student's *t*-test with Statistical Package for the Social Sciences (SPSS; v19.0). Postsurgery adverse events were assessed by the two-tailed chi-squared test. *P* < 0.05 indicated statistical significance.

## Results

Patient baseline features in both DiLEP and PKRP groups (Table 1) were not significantly different in any aspect. Perioperative indexes are summarized in Table 2. Hyponatremia was not observed in this study, and serum sodium decreases in both groups were similar (*P* = 0.380). In addition, weights of resected specimen were comparable between groups (*P* = 0.448). However, in comparison with PKRP treatment, the DiLEP group showed markedly reduced blood loss, shorter operation time, faster bladder irrigation and catheterization, and decreased hospitalization duration.

**Table 1.** Baseline Patient Characteristics

*Parameters*	*DiLEP (76)*	*PKRP (76)*	P
Age (years)	73.7 ± 8.4 (56–92)	71.5 ± 8.9 (55–93)	0.116
Prostate volume (mL)	56.2 ± 11.9 (32–80)	55.5 ± 13.1 (34–80)	0.746
PVR (mL)	204.6 ± 191.1 (20–700)	199.5 ± 186.8 (20–650)	0.867
Qmax (mL/second)	5.7 ± 2.2 (1.7–11.5)	6.2 ± 2.5 (2.5–13.6)	0.194
IPSS	24.5 ± 3.2 (18–30)	25.2 ± 3.0 (16–31)	0.164
QoL	4.7 ± 0.7 (3–6)	4.9 ± 0.7 (3–6)	0.115

Data are represented as mean ± SD for each parameter with *n* = 76.

DiLEP = diode laser enucleation of the prostate; IPSS = International Prostate Symptom Score; PKRP = plasmakinetic resection of the prostate; PVR = postvoid residual; Qmax = maximum flow rate; QoL = quality of life; SD = standard deviation.

**Table 2.** Perioperative Data

*Parameters*	*DiLEP (76)*	*PKRP (76)*	P
Operative time (minutes)	61.3 ± 19.0 (35–155)	94.5 ± 31.5 (35–180)	<0.001
Hemoglobin decrease (g/L)	9.5 ± 5.5 (1–25)	12.6 ± 7.2 (2–30)	0.004
Sodium decrease (mmol/L)	2.4 ± 1.7 (0–6)	2.2 ± 1.6 (0–8)	0.380
Resected weight (g)	34.8 ± 13.8 (10–65)	33.2 ± 12.7 (13–61)	0.448
Bladder irrigation time (hours)	15.9 ± 11.0 (0–83)	33.2 ± 21.2 (16–97)	<0.001
Catheter duration (days)	3.1 ± 1.2 (1–6)	5.5 ± 1.1 (3–8)	<0.001
Hospital stay (days)	7.9 ± 1.1 (5–10)	9.5 ± 1.1 (8–12)	<0.001

Data are represented as mean ± SD for each parameter with *n* = 76.

All 152 cases underwent follow-up assessment at 3, 6, and 12 months postoperation. Treatment outcomes are listed in Table 3. IPSS, QoL, Qmax, and PVR were comparable in both groups (*P* > 0.05).

**Table 3.** Data at Baseline and Clinical Outcomes at 3, 6, and 12 Months After Surgery

	*Baseline*	*3 Months*	*6 Months*	*12 Months*
IPSS				
DiLEP	24.5 ± 3.2 (18–30)	8.4 ± 2.8 (2–14)	6.8 ± 2.0 (2–12)	5.2 ± 1.9 (1–10)
PKRP	25.2 ± 3.0 (16–31)	8.7 ± 2.5 (3–15)	7.1 ± 2.1 (2–14)	5.4 ± 1.6 (2–10)
*P*	0.164	0.430	0.363	0.377
QoL				
DiLEP	4.7 ± 0.7 (3–6)	1.6 ± 0.7 (0–3)	1.4 ± 0.8 (0–3)	1.2 ± 0.7 (0–3)
PKRP	4.9 ± 0.7 (3–6)	1.8 ± 0.7 (0–3)	1.5 ± 0.8 (0–3)	1.4 ± 0.7 (0–3)
*P*	0.115	0.199	0.487	0.264
Qmax				
DiLEP	5.7 ± 2.2 (1.7–11.5)	17.6 ± 5.3 (7.3–37.3)	20.1 ± 3.9 (14.2–36.2)	20.7 ± 3.8 (15.3–37.5)
PKRP	6.2 ± 2.5 (2.5–13.6)	18.2 ± 4.8 (6.3–33.2)	21.1 ± 3.6 (15.1–33.0)	21.6 ± 3.7 (15.5–33.5)
*P*	0.194	0.475	0.123	0.144
PVR				
DiLEP	204.6 ± 191.1 (20–700)	18.6 ± 17.1 (0–50)	17.3 ± 12.4 (0–40)	11.6 ± 9.4 (0–30)
PKRP	199.5 ± 186.8 (20–650)	20.6 ± 17.1 (0–55)	18.8 ± 12.9 (0–35)	12.7 ± 11.0 (0–45)
*P*	0.867	0.478	0.463	0.527

Data are represented as mean ± SD for each parameter with *n* = 76.

No bladder injury, blood transfusion, and TURS cases were noted in this study. None of the cases had long-term urinary incontinence; five (6.6%) and three (3.9%) patients had transient incontinence in the DiLEP and PKRP groups, respectively. Secondary bleeding after 1 month was observed in two (2.6%) patients in the DiLEP group and in one (1.3%) patient in the PKRP group, respectively. Secondary urethral stricture after operation was observed in one (1.3%) and two (2.6%) patients in the DiLEP and PKRP groups, respectively. All complications in both groups are shown in Table 4.

**Table 4.** Perioperative Complications

*Complications*	*DiLEP (76)* n *(%)*	*PKRP (76)* n *(%)*	P
Intraoperative			
Blood transfusion	0	0	—
TURS	0	0	—
Capsule perforation	0	1 (1.3)	0.316
Bladder injury	0	0	—
Postoperative			
Secondary bleeding	2 (2.6)	1 (1.3)	0.560
Recatheterization	3 (3.9)	2 (2.6)	0.649
Transitory stress incontinence	5 (6.6)	3 (3.9)	0.468
Urethral stricture	1 (1.3)	2 (2.6)	0.560
Irritative symptoms	12 (15.8)	7 (9.2)	0.220

TURS = transurethral resection syndrome.

## Discussion

TURP remains the gold standard for treating BPH in male individuals with a prostate size between 30 and 80 mL.^12^ Despite the high success rate of TURP, there are concerns regarding perioperative morbidity and perioperative safety, especially related to bleeding.^1^ Indeed, TURP is not always safe, as cases with prostate volumes exceeding 80 mL and those with ongoing anticoagulation are totally contraindicated. Therefore, urologists make efforts to search for new endoscopic treatment options for patients with symptomatic BPH. Prostate artery embolization (PAE) is a new treatment for extremely enlarged BPH. PAE provides clinically and statistically significant improvement of symptoms and QoL.^13^ Aquablation is also a novel therapy using a high-velocity waterjet and real-time ultrasound imaging with robotic assistance for targeted removal of the prostate tissue.^14^ Transurethral enucleation of the prostate (TUEP) represents an endoscopic alternative for treating BPH in men, even for cases with large prostates.^15^ With development of laser devices, several laser types, including holmium, KTP, thulium, and diode lasers, have been adapted in TUEP. Holmium laser enucleation of the prostate (HoLEP) provides functional benefits lasting longer than those obtained with TURP or open prostatectomy in large prostates.^16,17^ However, HoLEP has a longer learning curve. Naspro and coworkers suggested >200 endoscopic surgeries are required for a surgeon to perform best.^18^ TUEP using a KTP laser or thulium laser is also efficient in symptomatic BPH.^19,20^

Recent technologic advances in laser vaporization of the prostate comprise the development of diode laser systems. Diode lasers have different wavelengths such as 940, 980, 1318, and 1470 nm. Evidence indicates that diode lasers constitute a novel, safe, and efficient technology for treating patients with BPH as TURP/TUEP. DiLEP is advantageous in that it provides markedly reduced blood loss, reduced hospital stay, and decreased catheter indwelling time.^21,22^

The current study showed that no patients required blood transfusion in either of the treatment groups. The procedure of DiLEP is associated with reduced intraoperative hemorrhage reflected by less pronounced postoperative hemoglobin level reduction in comparison with PKRP (9.5 ± 5.5 *vs* 12.6 ± 7.2, *P* = 0.004). The 1470 nm diode laser beam is absorbed by both water and hemoglobin, which results in excellent coagulation and rapid vaporization of the tissue. In the current study, surgical times for DiLEP and PKRP were 61.3 ± 19.0 and 94.5 ± 31.5 minutes, indicating a stark reduction in the former procedure (*P* < 0.001). DiLEP was faster in BPH treatment than PKRP, with a dramatically shortened operation time, which reduces surgical risks, especially in patients with cardiopulmonary insufficiency disease.

TURS is another severe complication of traditional TURP; however, no TURS case was observed in either of the treatment groups, with comparable serum sodium reductions in both groups. Because physiologic saline was used for irrigation, DiLEP and PKRP almost had no risk of TURS. In addition, rapid vaporization of the tissue and coagulation occurred almost at the same time during DiLEP for BPH, which to a great extent reduced the exposure time of blood vessels and subsequent odds of irrigation water entering into circulation.^23^

Transient stress urinary incontinence is often encountered after endosurgery for prostate enucleation, and was found in 3.3%–7.5% cases after DiLEP.^24^ This study demonstrated that DiLEP occurrence was 6.6%, compared with 3.9% in the PKRP group (*P* = 0.468), similar to HoLEP and thulium laser vaporization enucleation of the prostate (ThuVEP).^25,26^ Stress urinary incontinence disappeared at 3 months postoperatively by exercising the levator ani muscle. According to our experience, prostatic apexes are critical during DiLEP surgery for large prostates. Best efforts should be made to reduce injuries of the external sphincter during enucleation.

The incidence of irritative symptoms in DiLEP was 15.8%, with no significant difference in comparison with the PKRP group (15.8% *vs* 9.2%, *P* = 0.220). Such finding could be explained by use of the 1470 nm diode laser, which penetrates as deep as 2.3 mm, with elevated coagulation depth, as found in TURP.^27^ In contrast to other lasers, less coagulated and necrotic tissues after DiLEP are removed postoperatively.^21^ The incidence rates of recatheterization and urethral stricture after DiLEP were 3.9% and 1.3%, respectively, corroborating Razzaghi and coworkers,^28^ with both groups showing comparable values.

Accordingly, the curative effects of DiLEP and PKRP are similar; indeed, significant improvements were obtained in both groups from baseline values in IPSS, QoL score, Qmax, and PVR at postoperative 3, 6, and 12 months. However, both groups showed similar values for various assessment parameters at these time points. The present study suggested that DiLEP is as safe and efficient as PKRP.

This study had limitations because of the small sample size and short follow-up. Therefore, the current findings require confirmation in large prospective randomized trials.

## Conclusions

Both DiLEP and PKRP are safe and efficient for BPH, as shown in this initial study. In comparison with PKRP, DiLEP reduces the risk of hemorrhage, takes less time, decreases bladder irrigation and catheterization times, and results in decreased hospitalization duration. Further well-designed, prospective randomized studies with prolonged follow-up, including large numbers of patients, are required to confirm the benefits of DiLEP in symptomatic BPH.

## Abbreviations Used

BPH: benign prostatic hyperplasia
DiLEP: diode laser enucleation of the prostate
HoLEP: holmium laser enucleation of the prostate
IPSS: International Prostate Symptom Score
KTP: potassium-titanyl-phosphate
LUTS: lower urinary tract symptoms
PAE: prostate artery embolization
PKRP: plasmakinetic resection of the prostate
PSA: prostate-specific antigen
PVR: postvoid residual
Qmax: maximum flow rate
QoL: quality of life
RCT: randomized clinical trial
TRUS: transrectal ultrasonography
TUEP: transurethral enucleation of the prostate
TURP: transurethral resection of the prostate
TURS: transurethral resection syndrome
